# Supplementary material for: Metabolomic profiling reveals a differential role for hippocampal glutathione reductase in infantile memory formation
Source: eLife. 2021 Nov 26;10:e68590. doi: 10.7554/eLife.68590 (PMC8626085; doi:10.7554/eLife.68590)
Supplement: Figure 6—source data 2. [file elife-68590-fig6-data2.docx]

**Figures 6-8-Source Data 2**

| **Figure 6b** | **PN17** | **PN24** | **PN80** |  |
| --- | --- | --- | --- | --- |
| **GSH** | 116.07±4.84%  (n=7 rats) | 191.31±11.91%  (n=7 rats) | 100±10.99%  (n=7 rats) |  |
| One–way ANOVA followed by Tukey's multiple comparisons test  F (2, 18) = 24.92 ; P < 0.0001 | | | |  |
| **GSSG** | 138.13±16.58%  (n=7 rats) | 143.59±7.08%  (n=7 rats) | 100±4.79%  (n=7 rats) |  |
| One–way ANOVA followed by Tukey's multiple comparisons test  F (2, 18) = 4.864 ; P = 0.0205 | | | |  |
| **GSH/GSSG** | 90.79±8.93%  (n=7 rats) | 135.32±6.09%  (n=7 rats) | 100±7.17%  (n=7 rats) |  |
| One–way ANOVA followed by Tukey's multiple comparisons test  F (2, 18) = 9.857 ; P = 0.0013 | | | |  |
| **Glutamate** | 93.37±2.48%  (n=7 rats) | 122.39±2.53%  (n=7 rats) | 100±2.34%  (n=7 rats) |  |
| One–way ANOVA followed by Tukey's multiple comparisons test  F (2, 18) = 38.44 ; P < 0.0001 | | | |  |
| **Cysteine** | 26.97±3.59%  (n=7 rats) | 85.19±5.23%  (n=7 rats) | 100±9.77%  (n=7 rats) |  |
| One–way ANOVA followed by Tukey's multiple comparisons test  F (2, 18) = 32.96 ; P < 0.0001 | | | |  |
| **Glycine** | 53.57±1.66%  (n=7 rats) | 96.79±4.5%  (n=7 rats) | 100±4.53%  (n=7 rats) |  |
| One–way ANOVA followed by Tukey's multiple comparisons test  F (2, 18) = 46.36 ; P < 0.0001 | | | |  |

| **Figure 6c** | **PN17** | | **PN24** | **PN80** |  |
| --- | --- | --- | --- | --- | --- |
| **Glutathione reductase** | 76.92±3.59%  (n=6 rats) | | 84.57±4.58%  (n=4 rats) | 100±4.18%  (n=4 rats) |  |
| One–way ANOVA followed by Tukey's multiple comparisons test  F (2, 11) = 8.344 ; P = 0.0062 | | | | |  |
| **Glutathione peroxidase** | 196.3±9.68%  (n=6 rats) | | 155.22±9.74%  (n=4 rats) | 100±3.25%  (n=4 rats) |  |
| One–way ANOVA followed by Tukey's multiple comparisons test  F (2, 11) = 30.06 ; P < 0.0001 | | | | |  |
| **Glutamate-cysteine ligase - catalytic subunit** | 84.27±2.98%  (n=6 rats) | | 91.49±6.47%  (n=4 rats) | 100±5.07%  (n=4 rats) |  |
| One–way ANOVA followed by Tukey's multiple comparisons test  F (2, 11) = 3.046 ; P = 0.0886 | | | | |  |
| **Glutamate-cysteine ligase - regulatory subunit** | 36.62±5.41%  (n=6 rats) | 170.67±8.29%  (n=5 rats) | | 100±3.61%  (n=5 rats) |  |
| One–way ANOVA followed by Tukey's multiple comparisons test  F (2, 13) = 126.8 ; P < 0.0001 | | | | |  |
| **Glutathione synthetase** | 43.52±0.65%  (n=6 rats) | | 47.95±1.61%  (n=4 rats) | 100±3.58%  (n=4 rats) |  |
| One–way ANOVA followed by Tukey's multiple comparisons test  F (2, 11) = 240.0 ; P < 0.0001 | | | | |  |

| **Figure 6d** | **PN17** | | **PN24** | **PN80** |  |
| --- | --- | --- | --- | --- | --- |
| **Glutathione reductase activity** | 183.61±10.26%  (n=6 rats) | | 167.92±8.41%  (n=6 rats) | 100±9.38%  (n=6 rats) |  |
| One–way ANOVA followed by Tukey's multiple comparisons test  F (2, 15) = 22.44 ; P < 0.0001 | | | | |  |
| **Glutathione peroxidase activity** | 205.34±11.23%  (n=6 rats) | | 199.30±10.15%  (n=6 rats) | 100±8.11%  (n=6 rats) |  |
| One–way ANOVA followed by Tukey's multiple comparisons test  F (2, 15) = 35.59 ; P < 0.0001 | | | | |  |
| **Glutamate-cysteine ligase activity** | 43.88±6.59%  (n=6 rats) | | 172.65±13.83%  (n=6 rats) | 100±10.76%  (n=6 rats) |  |
| One–way ANOVA followed by Tukey's multiple comparisons test  F (2, 15) = 35.69 ; P < 0.0001 | | | | |  |
| **Glutathione synthetase activity** | 46.12±7.79%  (n=6 rats) | 86.99±7.69%  (n=6 rats) | | 100±11.11%  (n=6 rats) |  |
| One–way ANOVA followed by Tukey's multiple comparisons test  F (2, 15) = 9.751 ; P = 0.0019 | | | | |  |

|  |  | |  | |  | |  |  |  |  |
| --- | --- | --- | --- | --- | --- | --- | --- | --- | --- | --- |
| **Figure 7a** | | **PN17-N** | | **PN17-Tr** | | **PN24-N** | | **PN24-Tr** | **PN80-N** | **PN80-Tr** |
| **GSH** | | 100±4.17%  (n=7 rats) | | 86.12±7.15%  (n=7 rats) | | 100±6.22%  (n=7 rats) | | 100.34±5.34%  (n=7 rats) | 100±10.99%  (n=7 rats) | 124.75±12.58%  (n=7 rats) |
| Two–way ANOVA followed by Bonferroni’s multiple comparisons test  Interaction : F (2, 36) = 2.76022 ; P = 0.0767  Age : F (2, 36) = 2.76023 ; P = 0.0767  Group : F (1, 36) = 0.302648 ; P = 0.5856 | | | | | | | | | | |
| **GSSG** | | 100±12.0%  (n=7 rats) | | 57.92±5.45%  (n=7 rats) | | 100±4.93%  (n=7 rats) | | 99.26±2.58%  (n=7 rats) | 100±4.79%  (n=7 rats) | 111.40±5.79%  (n=7 rats) |
| Two–way ANOVA followed by Bonferroni’s multiple comparisons test  Interaction : F (2, 36) = 9.03248 ; P = 0.0007  Age : F (2, 36) = 9.03247 ; P = 0.0007  Group : F (1, 36) = 3.78125 ; P = 0.0597 | | | | | | | | | | |
| **GSH/GSSG** | | 100±9.83%  (n=7 rats) | | 141.09±5.8%  (n=7 rats) | | 100±4.49%  (n=7 rats) | | 100.66±4.37%  (n=7 rats) | 100±7.17%  (n=7 rats) | 112.34±8.06%  (n=7 rats) |
| Two–way ANOVA followed by Bonferroni’s multiple comparisons test  Interaction : F (2, 36) = 4.53042 ; P = 0.0176  Age : F (2, 36) = 4.53041 ; P = 0.0176  Group : F (1, 36) = 10.2027 ; P = 0.0029 | | | | | | | | | | |

| **Figure 7b** | **PN17-N** | **Tr-1h** | | **Tr-24h** | |
| --- | --- | --- | --- | --- | --- |
| **Glutathione reductase** | 100±5.61%  (n=6 rats) | 104.82±3.94%  (n=6 rats) | | 106.34±5.33%  (n=6 rats) | |
| One–way ANOVA followed by Tukey's multiple comparisons test  F (2, 15) = 0.4365 ; P = 0.6542 | | | | | |
| **Glutathione peroxidase** | 100±2.45%  (n=6 rats) | 103.12±4.69%  (n=6 rats) | | 103.48±4.92%  (n=5 rats) | |
| One–way ANOVA followed by Tukey's multiple comparisons test  F (2, 14) = 0.2232 ; P = 0.8028 | | | | | |
| **Glutamate-cysteine ligase - catalytic subunit** | 100±8.44%  (n=6 rats) | 101.02±10.05%  (n=6 rats) | | 89.17±6.72%  (n=5 rats) | |
| One–way ANOVA followed by Tukey's multiple comparisons test  F (2, 14) = 0.5274 ; P = 0.6014 | | | | | |
| **Glutamate-cysteine ligase - regulatory subunit** | 100±3.87%  (n=6 rats) | | 104.58±3.98%  (n=6 rats) | | 102.98±4.39%  (n=5 rats) |
| One–way ANOVA followed by Tukey's multiple comparisons test  F (2, 14) = 0.3452 ; P = 0.7139 | | | | | |
| **Glutathione synthetase** | 100±4.17%  (n=6 rats) | 107.90±4.32%  (n=6 rats) | | 107.91±4.08%  (n=5 rats) | |
| One–way ANOVA followed by Tukey's multiple comparisons test  F (2, 14) = 1.201 ; P = 0.3301 | | | | | |

|  |  | | |  | | | |  | | | |  |  | |  |  |  |
| --- | --- | --- | --- | --- | --- | --- | --- | --- | --- | --- | --- | --- | --- | --- | --- | --- | --- |
| **Figure 7c** | | **PN17-N** | | | **Tr-15min** | | | | **Tr-1h** | | | | **Tr-24h** | | **Tr-7d** | | **PN24-N** |
| **Glutathione reductase activity** | | 100±5.59%  (n=6 rats) | | | 150.88±4.06%  (n=6 rats) | | | | 125.66±8.7%  (n=6 rats) | | | | 124.49±5.99%  (n=6 rats) | | 77.06±6.95%  (n=6 rats) | | 70.17±8.51%  (n=6 rats) |
| One–way ANOVA followed by Dunnett's multiple comparisons test  F (5, 30) = 20.88 ; P < 0.0001 | | | | | | | | | | | | | | | | | |
| **Glutathione peroxidase activity** | | | 100±5.47%  (n=6 rats) | | | 98.52±5.49%  (n=6 rats) | | | 101.49±5.64%  (n=6 rats) | | | | 103.81±5.49%  (n=6 rats) | | |  | |
| One–way ANOVA followed by Dunnett's multiple comparisons test  F (3, 20) = 0.1668 ; P = 0.9175 | | | | | | | | | | | | | | | |  |  |
| **Glutamate-cysteine ligase activity** | | | 100±9.91%  (n=6 rats) | | | 97.28±11.31%  (n=6 rats) | | | | 104.28±8.16%  (n=6 rats) | | | | 100.72±9.08%  (n=6 rats) | |  |  |
| One–way ANOVA followed by Dunnett's multiple comparisons test  F (3, 20) = 0.08858 ; P = 0.9655 | | | | | | | | | | | | | | | |  |  |
| **Glutathione synthetase activity** | | | 100±16.89%  (n=6 rats) | | | | 99.78±9.65%  (n=6 rats) | | | | 111.98±10.85%  (n=6 rats) | | | 101.17±9.48%  (n=6 rats) | |  |  |
| One–way ANOVA followed by Dunnett's multiple comparisons test  F (3, 20) = 0.2347 ; P = 0.8711 | | | | | | | | | | | | | | | |  |  |

| **Figure 7d** | | | **PN17-N** | | | **SO-15min** | | | **Tr-15min** |  |  |
| --- | --- | --- | --- | --- | --- | --- | --- | --- | --- | --- | --- |
| **Glutathione reductase activity** | | | 100±9.34%  (n=6 rats) | | | 118.86±8.86%  (n=6 rats) | | | 168.66±14.02%  (n=6 rats) |  |  |
| One–way ANOVA followed by Tukey's multiple comparisons test  F (2, 15) = 10.42 ; P = 0.0015 | | | | | | | | | |  |  |
| \|  \|  \|  \| \|  \|  \|  \| \| --- \| --- \| --- \| --- \| --- \| --- \| --- \| \| **Figure 7e** \| **PN24-N** \| \| **Tr-15min** \| \| **Tr-1h** \| **Tr-24h** \|  \| \| **Glutathione reductase activity** \| 100±6.13%  (n=6 rats) \| \| 105.56±8.59%  (n=6 rats) \| \| 96.75±6.24%  (n=6 rats) \| 101.53±4.14%  (n=6 rats) \| \| One–way ANOVA followed by Dunnett's multiple comparisons test  F (3, 20) = 0.3193 ; P = 0.8113 \| \| \| \| \| \| \|  \|  \|  \|  \| \|  \| \|  \|  \| \| --- \| --- \| --- \| --- \| --- \| --- \| --- \| --- \| \| **Figure 7f** \| **PN80-N** \| \| **Tr-15min** \| \| **Tr-1h** \| \| **Tr-24h** \|  \| \| **Glutathione reductase activity** \| 100±9.38%  (n=6 rats) \| \| 106.56±11.69%  (n=6 rats) \| \| 99.23±14.79%  (n=6 rats) \| \| 90.83±11.22%  (n=6 rats) \| \| One–way ANOVA followed by Dunnett's multiple comparisons test  F (3, 20) = 0.2922 ; P = 0.8306 \| \| \| \| \| \| \| \| \|  \| \| \| \| \| \| \| \| \| | | | | | | | | | |  |  |
|  |  | | |  |  | |  | | |  |  |
| **Figure 7g** | **Neurons** | | | | **Astrocytes** | | | **Unlabeled** | |  |  |
| **NeuN** | 100±3.36%  (n=4 rats) | | | | 11.26±2.13%  (n=4 rats) | | | 19.26±2.71%  (n=4 rats) | |  |  |
| One–way ANOVA followed by Tukey's multiple comparisons test  F (2, 9) = 312.1 ; P < 0.0001 | | | | | | | | | |  |  |
| **GFAP** | | 14.83±3.41%  (n=4 rats) | | | 100±6.75%  (n=4 rats) | | | 22.92±2.45%  (n=4 rats) | |  |  |
| One–way ANOVA followed by Tukey's multiple comparisons test  F (2, 9) = 105.0 ; P < 0.0001 | | | | | | | | | |  |  |

| **Figure 7g** | **Glutathione reductase activity** (nmol/min/mg prot) | | |
| --- | --- | --- | --- |
| **Cell-types** | **Neurons** | **Astrocytes** | **Unlabeled** |
| PN17-N (n=5) | 62.42±4.17 | 38.08±4.17 | 59.54±4.81 |
| Tr-15 min (n=5) | 97.61±8.69 | 46.76±5.17 | 77.85±9.25 |
| Two–way ANOVA followed by Bonferroni’s multiple comparisons Test  Interaction: F (2, 24) = 2.197 ; P = 0.1330  Group: F (1, 24) = 15.73 ; P = 0.0006  Cell-type: F (2, 24) = 18.17 ; P < 0.0001 | | | |

| **Figure 8a** | **N-Veh** | **N-2-AAPA 100uM** | **N-2-AAPA 200uM** | **Tr-Veh** | **Tr-2-AAPA 100uM** | **Tr-2-AAPA 200uM** |  |
| --- | --- | --- | --- | --- | --- | --- | --- |
| **Glutathione reductase activity** | 100±5.05%  (n=5 rats) | 60.36±8.11%  (n=5 rats) | 22.86±4.77%  (n=5 rats) | 146.73±12.92%  (n=5 rats) | 109.94±9.62%  (n=5 rats) | 25.51±4.41%  (n=5 rats) |  |
| One–way ANOVA followed by Tukey's multiple comparisons test  F (5, 24) = 37.66 ; P < 0.0001 | | | | | | |  |

| **Figure 8b** | **Mean Latency (s)** | | | |
| --- | --- | --- | --- | --- |
| **PN17** | **Acq** | **T1** | **T2/Tr** | **T3** |
| Vehicle (n=8) | 20.82±5.22 | 34.77±13.85 | 460.24±59.96 | - |
| 2-AAPA (100 uM) (n=6) | 12.81±3.07 | 26.34±14.49 | 113.49±34.59 | 601.04±56.41 |
| 2-AAPA (200 uM) (n=7) | 27.77±8.68 | 88.11±21.01 | 72.94±18.57 | 673.54±71.86 |
| Two–way RM ANOVA followed by Bonferroni’s multiple comparisons test  Interaction: F (4, 36) = 27.66 ; P < 0.0001  Timepoint: F (2, 36) = 50.95 ; P < 0.0001  Treatment: F (2, 18) = 13.57 ; P = 0.0003 | | | | |

| **Figure 8c** | **Mean Latency (s)** | | |
| --- | --- | --- | --- |
| **PN24** | **Acq** | **T1** | **T2** |
| Vehicle (n=7) | 15.03±3.68 | 545.82±66.76 | 525.25±55.29 |
| 2-AAPA (100 uM) (n=7) | 18.65±6.44 | 491.96±78.04 | 450.23±75.11 |
| Two–way RM ANOVA followed by Bonferroni’s multiple comparisons Test  Interaction: F (2, 24) = 0.3076 ; P = 0.7380  Timepoint: F (2, 24) = 58.79 ; P < 0.0001  Treatment: F (1, 12) = 0.6129 ; P = 0.4489 | | | |

| **Figure 8d** | **Mean Latency (s)** | | |
| --- | --- | --- | --- |
| **PN80** | **Acq** | **T1** | **T2** |
| Vehicle (n=6) | 15.44±4.33 | 420.2±58.62 | 498.27±50.07 |
| 2-AAPA (100 uM) (n=6) | 16.44±4.29 | 443.09±72.48 | 449.48±50.76 |
| Two–way RM ANOVA followed by Bonferroni’s multiple comparisons Test  Interaction: F (2, 20) = 0.03153 ; P = 0.9690  Timepoint: F (2, 20) = 56.47 ; P < 0.0001  Treatment: F (1, 10) = 0.08992 ; P = 0.7704 | | | |

| **Figure 8e** | **GSH** | | | |
| --- | --- | --- | --- | --- |
| **Timepoint** | **PN17-N** | **Tr-15 min** | **Tr-1h** | **Tr-24h** |
| Vehicle (n=6) | 100±5.16% | 146.41±7.26% | 97.09±3.83% | 94.85±6.58% |
| 2-AAPA (100 uM) (n=6) | 46.69±5.22% | 98.83±5.84% | 95.44±5.97% | 104.27±5.08% |
| Two–way ANOVA followed by Bonferroni’s multiple comparisons test  Interaction: F (3, 40) = 15.53 ; P < 0.0001  Timepoint: F (3, 40) = 25.01 ; P < 0.0001  Treatment: F (1, 40) = 33.33 ; P < 0.0001 | | | | |

| **Figure 8f** | **GSSG** | | | |
| --- | --- | --- | --- | --- |
| **Timepoint** | **PN17-N** | **Tr-15 min** | **Tr-1h** | **Tr-24h** |
| Vehicle (n=6) | 100±11.27% | 53.52±5.34% | 53.71±5.59% | 54.29±3.82% |
| 2-AAPA (100 uM) (n=6) | 161.13±9.72% | 106.25±14.15% | 108.98±10.60% | 99.80±7.21% |
| Two–way ANOVA followed by Bonferroni’s multiple comparisons test  Interaction: F (3, 40) = 0.2538 ; P = 0.8582  Timepoint: F (3, 40) = 15.91 ; P < 0.0001  Treatment: F (1, 40) = 69.73 ; P < 0.0001 | | | | |

| **Figure 8g** | **GSH/GSSG** | | | |
| --- | --- | --- | --- | --- |
| **Timepoint** | **PN17-N** | **Tr-15 min** | **Tr-1h** | **Tr-24h** |
| Vehicle (n=6) | 100±13.99% | 262.25±21.44% | 172.96±12.34% | 166.44±17.78% |
| 2-AAPA (100 uM) (n=6) | 27.38±3.44% | 94.93±13.94% | 85.39±11.04% | 100.19±11.57% |
| Two–way ANOVA followed by Bonferroni’s multiple comparisons test  Interaction: F (3, 40) = 5.520 ; P = 0.0029  Timepoint: F (3, 40) = 22.57 ; P < 0.0001  Treatment: F (1, 40) = 97.76 ; P < 0.0001 | | | | |
